# Mechanism and antibacterial activity of vine tea extract and dihydromyricetin against *Staphylococcus aureus*

**DOI:** 10.1038/s41598-020-78379-y

**Published:** 2020-12-08

**Authors:** Haiyun Liang, Keke He, Ting Li, Shumei Cui, Meng Tang, Shaoyi Kang, Wei Ma, Liya Song

**Affiliations:** 1 Beijing Key Laboratory of Plant Resources Research and Development, School of Science, Beijing Technology and Business University, No. 11 Fucheng Road, Haidian District, Beijing, 100048 China; 2 Key Laboratory of Cosmetic of China National Light Industry, School of Science, Beijing Technology and Business University, Beijing, China

**Keywords:** Microbiology techniques, Biomaterials

## Abstract

Vine tea (*Ampelopsis grossedentata)* has been approved as a new food ingredient in 2013. Both vine tea extract (VTE) and its active ingredient, 2R, 3R-Dihydromyricetin (DMY), showed good antibacterial activity. The mechanism of VTE and DMY against *Staphylococcus aureus* were evaluated by morphology observation, cell membrane and wall assay, protein assay, and DNA assay in this study. The results of SEM and TEM revealed that the VTE and DMY changed the morphology of *S. aureus*. The leakage of AKPase and β-galactosidase in treated groups demonstrated that the membrane integrity of *S. aureus* was disrupted. Meanwhile, the results of protein assay showed that VTE and DMY inhibited the expression of total proteins, and decreased activities of a few energy metabolism enzymes, total ATPase. Moreover, spectral and competitive analysis revealed that VTE and DMY interacted with DNA by groove and intercalation binding. Finally, the suspension experiments of Chinese cabbage and barley showed that inhibitors had strong inhibitory effect on bacteria growth. Overall, the results suggested that VTE and DMY may be potential food preservatives for inhibiting pathogen.

## Introduction

As reported by the World Health Organization (WHO), about 420,000 people die from food poisoning worldwide each year. *Staphylococcus aureus* is a common foodborne pathogen, which is considered to be the main dangerous pathogenic bacteria contributed to food poisoning^1^. *Staphylococcus aureus* can grow in many food and other products (such as cosmetic), and cause contamination for its tenacious survival ability in potentially dry and stressful environments^2,3^. Therefore, the food and some other industries have used additives to diminish microbial growth or inhibit microorganisms to avoid food contamination^4^. Recently, researchers toward the development of natural, safe and effective antimicrobial agents as increasing inecological and health risks associated with synthetic antimicrobials motivates^5^. In recent years, there have been many studies indicated that plant extracts, such as phenols and flavonoids, showed good antibacterial activities on bacterial^6^. Therefore, development of plant-derived compounds with antibacterial activities is one of the ways to solve the current problems.

**Figure 1:**
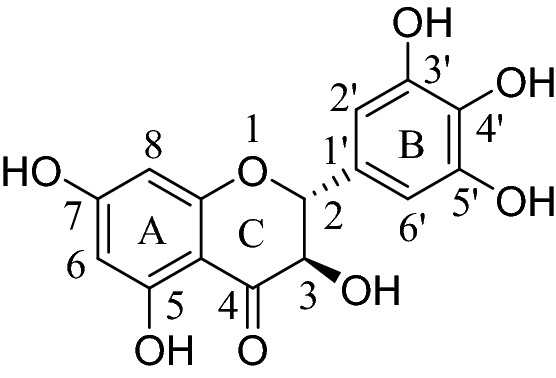
The structural of DMY (Version number: ChemOffice Professional 19, URL link: https://www.chemdraw.com.cn/product.html).

Plant extracts are still widely used in China for their medical treatment and health function. Chinese vine tea (*Ampelopsis grossedentata*), a plant belonging to Vitaceae family and *Ampelopsis Michx*, is distributed widely among mountainous areas of southern China. Chinese vine tea extracts (VTE) had been widely reported on its antibacterial, antioxidant, antihypertensive effect had been widely reported^7^. And dihydromyricetin (DMY) is the most abundant flavonoids of vine tea, which carries a lot of biological activities^8^. The main structure of DMY was shown in Fig. 1. The amount of DMY in Chinese vine tea varied from 20 to 93.1% according to different extracting conditions^9^. Xiao found that the mixture of DMY and traditional preservatives such as potassium sorbate and sodium benzoate could prolong the shelf life of the food^10^. Our study also showed that VTE and DMY have antibacterial inhibitory effect in *Staphylococcus aureus*, and they possess good potential as food additives. However, the research on the antibacterial mechanism of VTE and DMY is not very clear, which limits their application.

In order to provide a theoretical basis of the further application of VYE and DMY in food, the antibacterial mechanism of VTE and DMY against *Staphylococcus aureus* were systematically evaluated in this study, including of the growth curves of *S.aureus,* electron microscope experiments, energy metabolism enzyme experiments and DNA binding experiments, etc.

## Materials and methods

### Materials and reagents

*Staphylococcus aureus* ATCC 6538 was obtained from the China Medical Culture Collection Center (Beijing, China). The newly germinated spear leaves of *Ampelopsis grossedentata* were fermented and picked in Zhangjiajie City, Hunan Province at May 2019. The dried and pulverized vine tea (10.0 g) was extracted by deionized water (100 mL) at 100 °C for 1 h. The combined filtrates were concentrated on 20 mL by Rotary Evaporator, which were stored at 4 °C for further experiments. DMY (purity ≥ 90%) was obtained from Beijing Banxia Biology Technology Co, Ltd (Beijing, China). The AKP enzyme Assay Kit was purchased from Jiancheng Institute of bioengineering (Nanjing, China). Another chemicals and reagents used were of analytical grade, such as Na_2_CO_3_, analytical-grade anhydrous ethanol, methanol, nitrobenzene β-d-galactoside and etc. Nutrient broth and nutrient agar were purchased from Beijing Aobox Biotechnology (Beijing, China).

### The HPLC analysis of VTE and DMY

An HPLC system (Agilent Technologies, 1260 Infinity, USA) was used for the analytical characterization of the final VTE products. A C18 column (4.6 mm × 250 mm, 5 μm pore size, Agilent, USA) was eluted with methanol/water (with 0.03% phosphoric acid) (35:65) at a flow rate of 0.5 mL/min and temperature of 40℃. The injection volume was 5 μm, and the effluent was monitored at 292 nm with a Diode-array detector. The DMY (90% of HPLC purity) was obtained from Beijing Enokai Biotechnology Co., Ltd. (Beijing, China).

### Detection of minimum inhibitory concentration (MIC)

The experiment was carried out according to the reported method^11^. In brief, *S. aureus*, which was in the logarithmic growth phase of, was inoculated into nutrient broth medium (bacteria suspension concentration is 1 × 10^6^ CFU/mL). VTE (or DMY) were added to nutrient broth medium, giving final concentrations of 0.16 to 50 mg/mL. The *S. aureus* culture containing aseptic distilled water without VTE (or DMY) was the control. Bacterial suspension (100 μL) was plated onto the nutrient agar plates and incubated for 24 h in a 37 °C incubator. Bacterial growth was observed, and the minimum inhibitory concentration (MIC) was determined as the smallest dilution of VTE (or DMY) in which bacteria did not grow in nutrient agar plates.

### Growth curves

The measurement of *S. aureus* growth curve was determined according to a modified method^12^. The activated *S. aureus* were inoculated in 100 mL nutrient broths which was treated with different VTE (or DMY) concentration of 1/4 × MIC, 1/2 × MIC and 3/4 × MIC. The *S. aureus* culture containing aseptic distilled water without VTE (or DMY) was the control. Then, the culture medium was incubated under the condition of 37 °C and 120 r/min. The Growth curves drawn by optical density (OD_540) at 0, 2, 4, 6, 8, 10, 22, 30 h.

### Antibacterial activity in food systems

The antibacterial activity in food systems experiments were carried out as previously reported with minor modifications^13^. Briefly, cabbage suspension consists of squeezing cabbage juice and aseptic distilled water (50% vol/vol). Barley soup is made up of pulverized barley powder and aseptic distilled water (5% wt/vol). The food model media can be used after high pressure sterilization. 0.1% Tween 80 was added into cabbage suspension and barley soup for emulsion stabilization. While *S. aureus* was inoculated into the food model media (bacteria suspension concentration is 1 × 10^6^ CFU/mL), VTE (or DMY) were added to the food model media, giving final concentrations of 1/2 × MIC, 1 × MIC at 37 °C for 9 days. Moreover, quantification of viable cells was obtained by diluting sample suspensions and spreading on the nutrient agar medium at 37 °C for 24 h.

### Membrane integrity

#### Extracellular alkaline phosphatase (AKPase) assay

Bacterial cells of *S.aureus* were treated with 1 × MIC VTE (or DMY ) for 12 h and washed three times using 0.1 mol/L PBS (pH 7.2), a group with no treatment served as control. After that, bacterial suspensions were centrifuged at 10,000 rpm for 2 min at 4 °C in a highspeed centrifuge. Cells were then resuspended in the PBS buffer and adjusted to the same optical density (OD_420) value at 37 °C water bath. The measurement was performed with commercial kits from Nanjing Jiancheng Institute according to the manufacturer's instructions.

#### Extracellular β-galactosidase assay

Bacterial cells of *S.aureus* processing procedure was same as above steps (2.5.1), Nitrobenzene β-d-galactoside (30 mmol/L) and bacteria suspension were mixed in the ratio of 2:19 for 30 s. With water-bath heating reaction to 37 °C, 210 uL Na_2_CO_3_ (30 mmol/L) as elimination agent, 2 mL bacteria culture was removed from the mixture and was centrifuged at 0, 1, 2, 3, 4 h. Finally, supernatant optical density was measured with a microplate reader (Infinite M200PRO, TECAN, Männedorf, Switzerland) at OD_416.

### Scanning electron microscopy and transmission electron microscopy

The scanning electron microscope (Quanta 200, FEI, USA) analysis was done to observe the changes which occurred in the cell surface of bacterial after treatment of 1MIC DMY or VTE. Bacterial cells after treatment of DMY or VTE for 12, 24 h were centrifuged at 8000 rpm for 8 min to pellet down. The total bacterial cells were washed twice a time of phosphate buffer saline. Washed bacterial pellets were fixed in 3.0% glutaraldehyde for 48 h and again washed with phosphate buffer saline 10 times. The bacterial pellets were immersed in 1% osmic acid for 2 h, then the total bacterial cells were washed in 30 min with double distilled water. Following washing, bacterial pellets were washed with graded ethanol (30%, 50%, 70%, 80%, 90%, 95%, 100%) and every eluting time was 15 min. Isoamyl acetate was used to replace ethanol as intermediate medium in samples. The sample preparation for emission scanning electron microscopy was done as per instruction with few modifications.

The transmission electron microscope analysis was done to observe the changes which occurred in the cytoplasm, cell membrane of bacterial after treatment of 1 × MIC DMY or VTE. The pretreatment of bacterial was same as the process of scanning electron microscopy. After routine steps of desiccation and clearing, anhydrous acetone was used to replace ethanol as intermediate medium in samples.

### Energy metabolism enzyme and protein expression assay

#### Intracellular protein expression in bacteria

The pretreating methods of bacteria were the same as described in 2.5.1. Microbiologicalintracellular proteins in cell suspension were detected by the method described by Ting Li and others^14^.

#### Analysis of bacteria proteins by SDS-PAGE

The experiment was carried out according to the reported method^15^. Stacking gel (4%) and SDS–polyacrylamide gel (10%) were used for SDS-PAGE analysis. *S. aureus* in the logarithmic phase was added into the liquid medium containing VTE or DMY (1 × MIC), and the final bacterial concentration was 1 × 10^7^ CFU/mL. Samples were cultured for 6 and 24 h at 37 °C. All samples were centrifuged at 14,000 rpm for 5 min to pellet down. and washed thrice with sterile phosphate buffer (PBS). The collected bacteria were diluted into the same concentration determined by the OD_480 values. The samples were denatured for 5 min in 4 × SDS-PAGE loading buffer, and then supernatant was loaded on the gel for SDS-PAGE analysis.

#### Key enzymes assay in energy metabolism process of bacteria

The pre-manufacture procedures of bacteria were the same as described in 2.6.1. Cell suspension was centrifuged and the supernatant was got. The succinate dehydrogenase activity, Malate dehydrogenase activity and total adenosine triphosphate (ATPase) activity were assayed according to directions of test kit from Nanjing Jiancheng Institute (Nanjing, China).

### DNA binding assay

#### UV spectroscopy

Different concentrations of *S. aureus* genomic DNA (0–0.6 mg/mL) were added with 100 μL concentration of VTE or DMY (0.6 mg/mL), respectively. They were then incubated at 37 °C for 1 h with a UV scanning range of 200 to 450 nm.

#### Fluorescence spectroscopy

The VTE or DMY and genomic DNA were treated in the same way as described in 2.8.1. After one hour of incubation, it was scanned by fluorescence spectrophotometer with an excitation wavelength of 437 nm, and the emission wavelength range is 400 to 650 nm.

### Statistical analysis

All assays were performed intriplicates and the results are the means of three independent experiments. The data were analyzed by one-way Analysis of Variance (ANOVA) using SPSS software. The data are expressed as the means ± standard deviation (S.D.) of at least three independent experiments. Significance was determined at P < 0.05.

## Results

### The HPLC chromatogram of VTE and DMY

**Figure 2:**
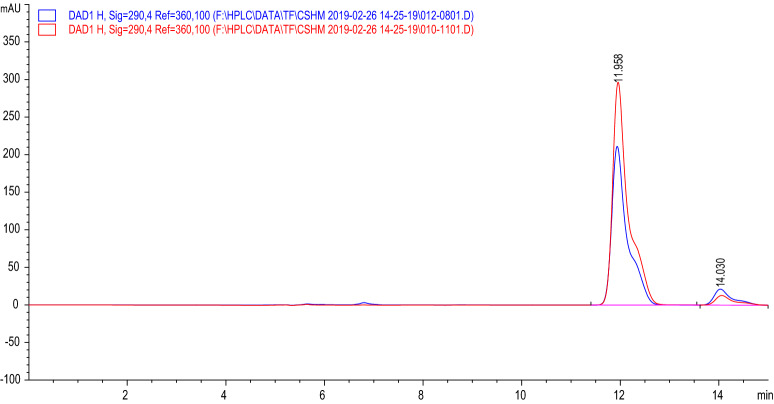
The HPLC of VTE (14.030 min) and DMY (11.958 min).

As shown in Fig. 2, it is a determination of the content of DMY in VTE by HPLC. The standard curve fitting equation of DMY standard is y = 66478x + 7.3095, R^2^ = 0.9991(The X is the DMY concentration (g/L), and the Y is the peak area). The final result is that the concentration of DMY is about 65.29%.

### Establishment of the MIC

The MIC of VTE、DMY were 6.3 mg/ mL and 1.25 mg/mL, respectively.

### Effect of VTE and DMY on the growth of *S. aureus*

#### Effects on cell growth curve

**Figure 3:**
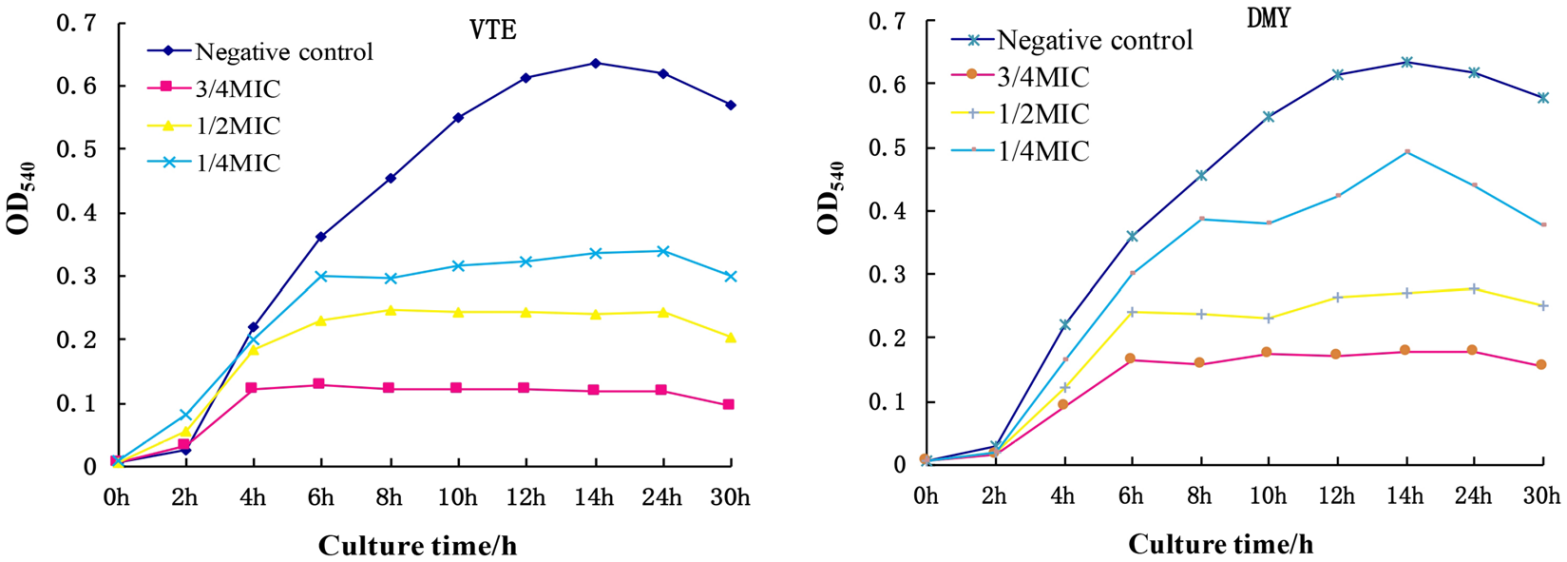
The effect of inhibitors on the growth Curve of *S. aureus*.

The effect of VTE and DMY on the growth curves of *S. aureus* were shown in Fig. 3. Compared with the control group, the optical density of the bacterial solution treated with the VTE and DMY were significantly decreased. From the changes of the growth curve, the stagnation period of cell growth was prolonged by VTE and DMY, the logarithmic phase of *S. aureus* was obviously limited, and the number of bacteria decreased obviously. In addition, the inhibitory effect was enhanced with the increase in inhibitor concentration of a certain range.

#### Effect on morphology of *S. aureus*

**Figure 4:**
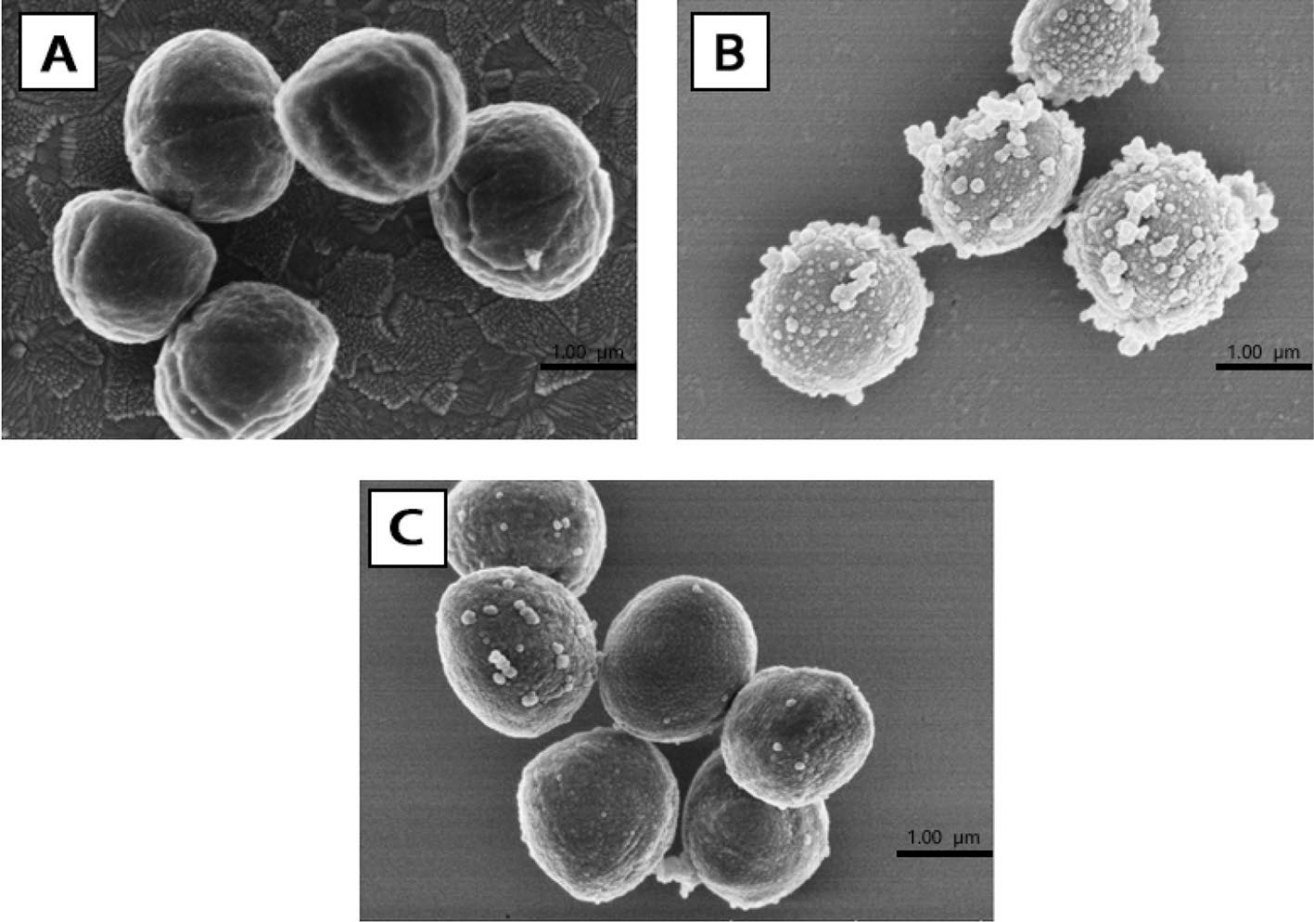
Morphological structure of *S. aureus* observed by SEM, untreated (**A**); VTE treatment 12 h (**B**); DMY treatment 12 h (**C**).

The effects of VTE and DMY on the morphologies of *S. aureus* cells were visualized by SEM (Fig. 4). In control group, the cell is smooth, full appearance and good refraction (Fig. 4A). After VTE treatment for 12 h, there were obvious vesicular and irregular processes on the surface of *S. aureus* (Fig. 4B). There was a large amount of dissolution around *S. aureus* after treatment of DMY for 12 h (Fig. 4C), the contents of the outflow may be intracellular materials. In addition, as shown in the images, the effect of VTE on the external morphology of microorganisms is more obvious than that of DMY.

**Figure 5:**
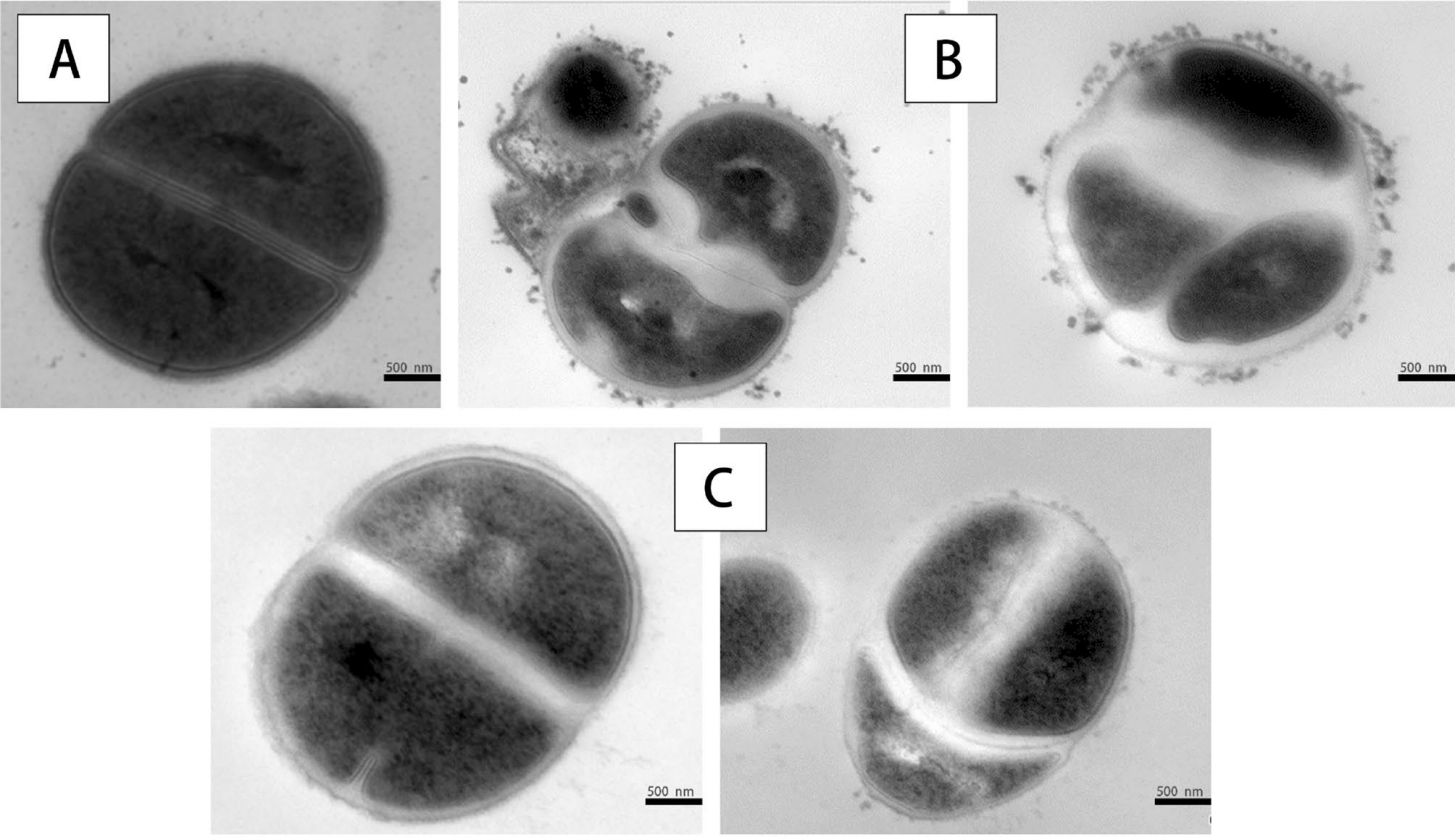
Morphological structure of *S. aureus* observed by TEM, untreated (**A**); VTE treatment 12 h (**B**); DMY treatment 12 h (**C**).

The TEM of *S. aureus* is shown in Fig. 5. *S. aureus* cell wall and membrane were seriously damaged by VTE for 12 h (Fig. 5B) showing the cell boundary becomes blurred, the extracellular solutes existed and the bacteria autolysis. Additional, the cytoplasm became lighter and fragile, and the nuclear area also changed obviously, such as the decrease of nuclear quantity. However, compared with VTE group, the phenomenon of DMY treatment group was not very obvious (Fig. 5C). Finally, *S. aureus* was blurred in cell division after treatment of VTE and DMY.

### Effects of VTE and DMY on the cell wall and membrane

#### Effect on membrane integrity

**Figure 6:**
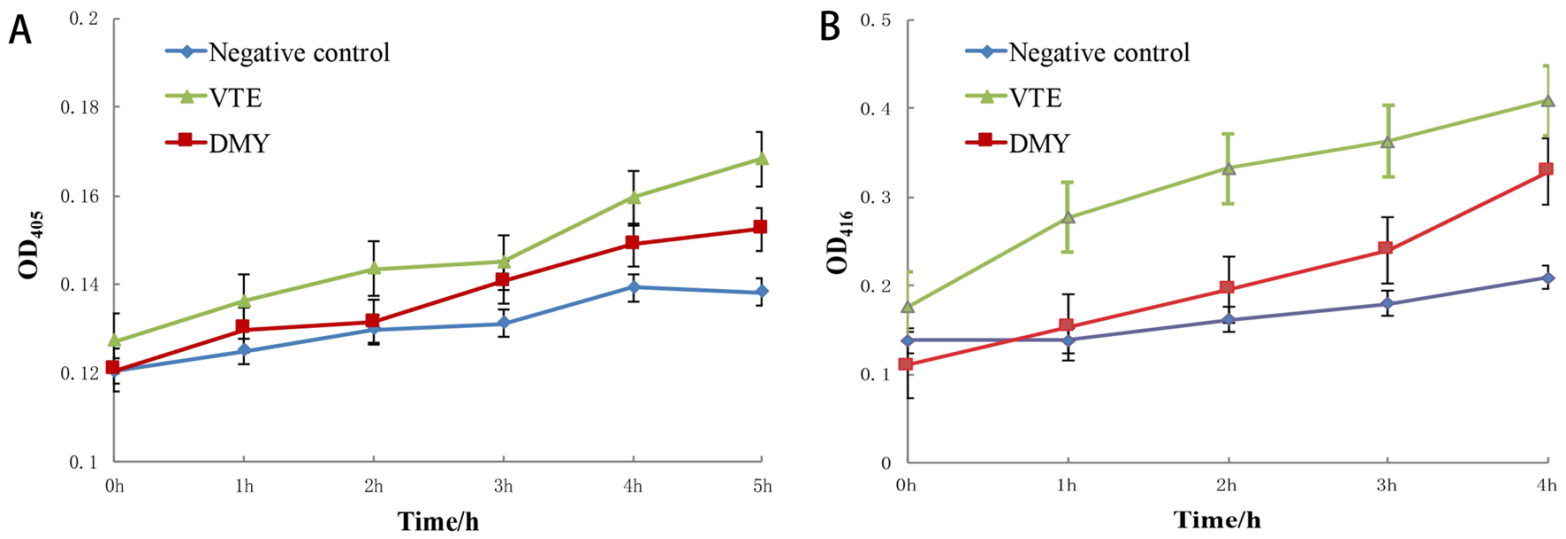
Changes of extracellular AKPase content of *S. aureus* after 12 h of treatment with VTE and DMY (**A**); Changes of extracellular β-galactosidase content in *S. aureus* treated with VTE and DMY for 12 h (**B**).

Alkaline phosphatase (AKPase) is located between the cell wall and membrane. A large amount of intracellular AKP leakage can indicate that the integrity of the cell wall has been destroyed. The AKPase released treated with 1 × MIC VTE or DMY for 12 h was shown in Fig. 6A, the extracellular AKPase of VTE and DMY treated groups increased by 21.8% and 10.3%, respectively. The extracellular AKPase treated with VTE and DMY was significantly higher than that of the control group, suggesting the VTE and DMY destroyed the integrity of the cell wall.

#### Effect on cell membrane permeability

The changes of extracellular β-galactosidase contents were shown in Fig. 6B. The content of extracellular β-galactosidase treated with VTE and DMY were increased by 90.2% and 57.4%, respectively, which were significantly higher than that in the control group. The results indicated cell membrane permeability was destroyed by VTE and DMY.

### Effects of VTE and DMY on *S. aureus* protein

#### Effect on the total protein expression

**Figure 7:**
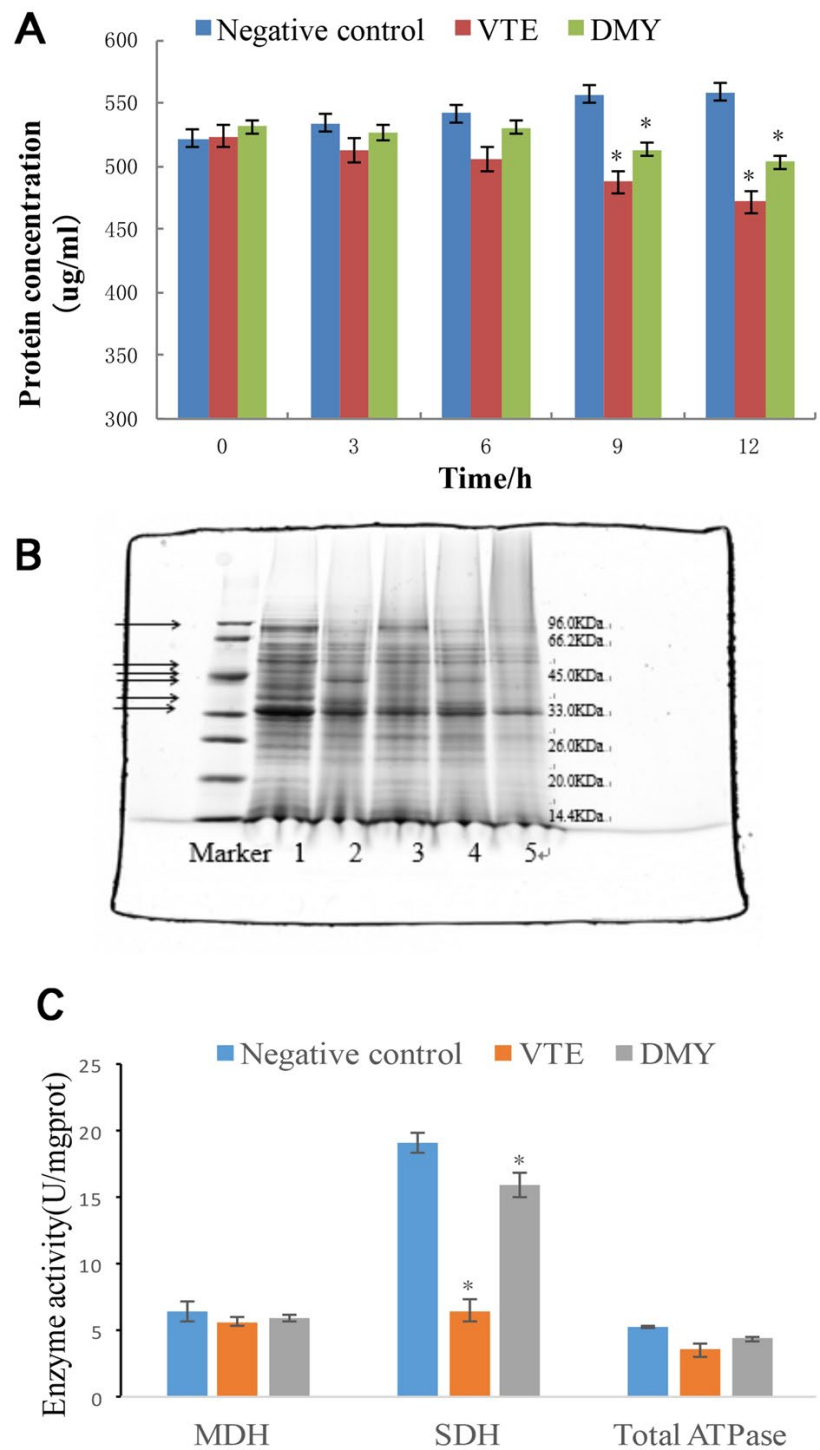
Changes of intracellular protein content in *S. aureus* treated with inhibitor (**A**). SDS-PAGE electrophoresis diagram of *S. aureus* under the action of inhibitor: 1. Negative control; 2. DMY-6 h; 3. DMY-24 h; 4. VTE-6 h; 5. VTE-24 h (**B**). Activity of key enzymes in Energy Metabolism of *S. aureus* treated with Inhibitors for 12 h (**C**).

Protein plays a key role in bacterial metabolic activity. The changes of intracellular protein content in cells treated with VTE and DMY are shown in Fig. 7A. The results showed that the content of soluble protein in the control group was basically stable and increased slowly with time, while the contents of soluble protein in *S. aureus* treated with VTE and DMY were decreased to varying degrees. The total protein were decreased with the increase of treatment time, the total protein content in VTE group and DMY group decreased by 15.5% and 9.9% (P < 0.05), respectively, indicating the VTE and DMY inhibited the protein synthesis.

#### SDS-PAGE analysis

The results of SDS-PAGE analysis of *S.aureus* under the action of DMY and VTE were shown in Fig. 7B. At 85.0 KDa and 55.0–33.0 KDa, the color of the protein strip became lighter with the increase of treated time. In addition, there were protein bands disappeared or appeared. Compared with DMY, the change of band color after VTE treatment was more obvious, and the change of protein band was the biggest after 24 h treatment. Therefore, it is speculated that DMY and VTE have effects and differences on the expressions of bacterial proteins, so a few metabolic enzymes were picked for the next step.

#### Effect on energy metabolism enzyme

Activities of key enzymes in energy metabolism of *S. aureus* treated with inhibitors for 12 h were shown in Fig. 7C. Compared with the control group, the activities of MDH, SDH and total ATPase of *S. aureus* treated with VTE for 12 h were decreased by 12.7%, 65.8% and 31.5%, and the activities of the three enzymes treated with DMY decreased by 4.7%, 16.7% and 15.9%, respectively. Thus, the VTE had stronger effect on the three key enzymes in energy metabolism, especially on SDH.

### Effects of VTE and DMY on the nuclear DNA

#### UV spectroscopy studies

**Figure 8:**
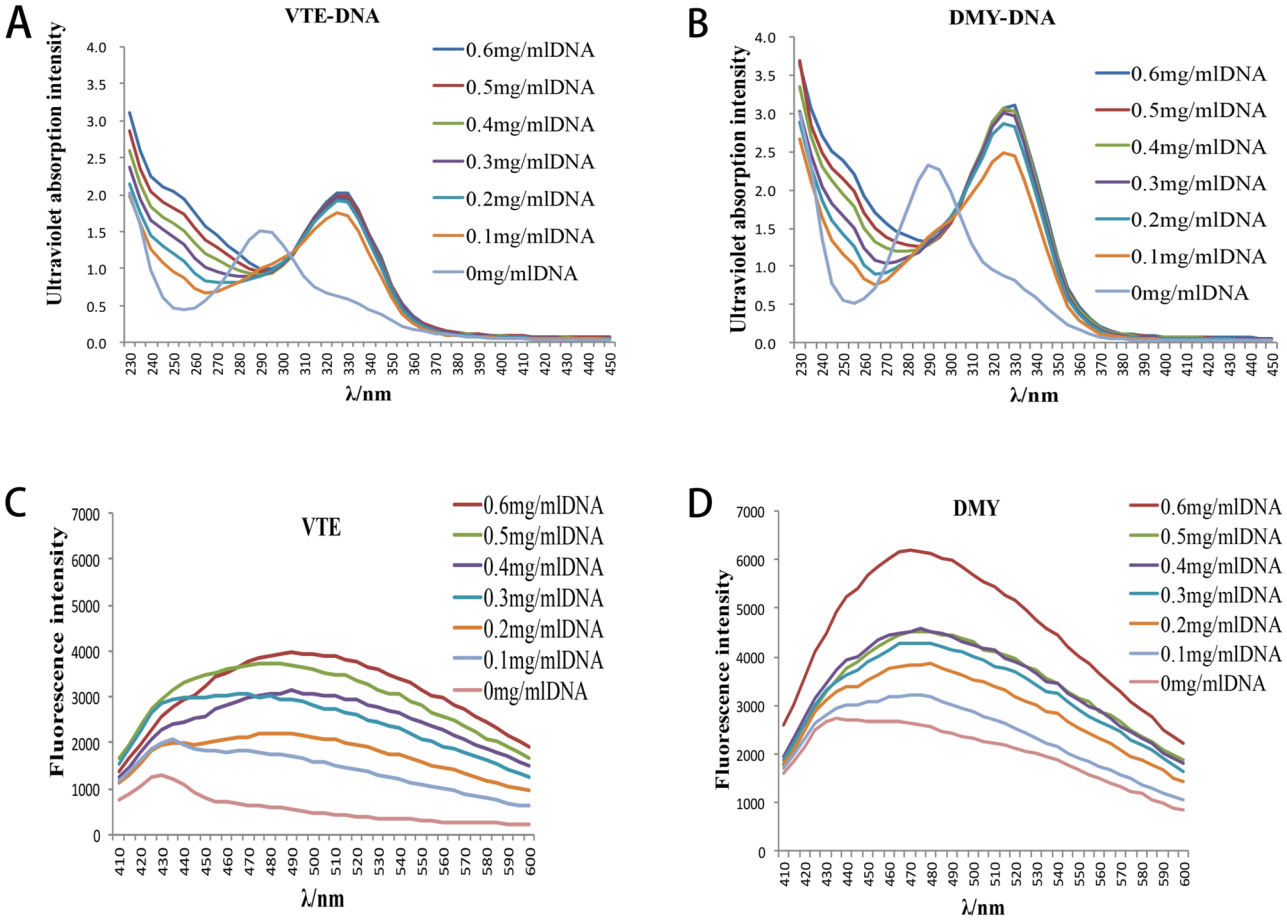
Effect of *S. aureus* Genomic DNA on Ultraviolet Spectra of VTE and DMY (**A**,**B**). Effect of *S. aureus* Genomic DNA on fluorescence Spectra of VTE and DMY (**C**,**D**).

The UV–vis absorption spectra of VTE and DMY varying with the concentration of bacterial genomic DNA are shown in Fig. 8A,B. Obvious redshift occurred (290–325 nm) when 0.1 mg/mL bacterial genomic DNA was added. and the absorbance was increased with the increasing of VTE and DMY, indicating that the VTE and DMY can interact with DNA^16^. In addition, when the concentration of bacterial genomic DNA was more than 0.2 mg/mL, the absorbance of VTE and DMY did not increase with the increase of DNA concentration, indicating there may be more than one type of binding between VTE/DMY and DNA. So the specific binding between inhibitors and DNA need to be further studied.

#### Fluorescence studies

The fluorescence spectra of different concentrations of bacterial genomic DNA with VTE or DMY were obtained by fluorescence scanning, as shown in Fig. 8C,D. In the presence of *S. aureus* genomic DNA, the maximum emission wavelengths of VTE and DMY were also red-shifted, and the fluorescence intensities of VTE and DMY were significantly enhanced, resulting in an obvious hyperchromic effect. Therefore, it is inferred that VTE and DMY may have the effect of embedding DNA base pairs^16^.

#### Competitive analysis

**Figure 9:**
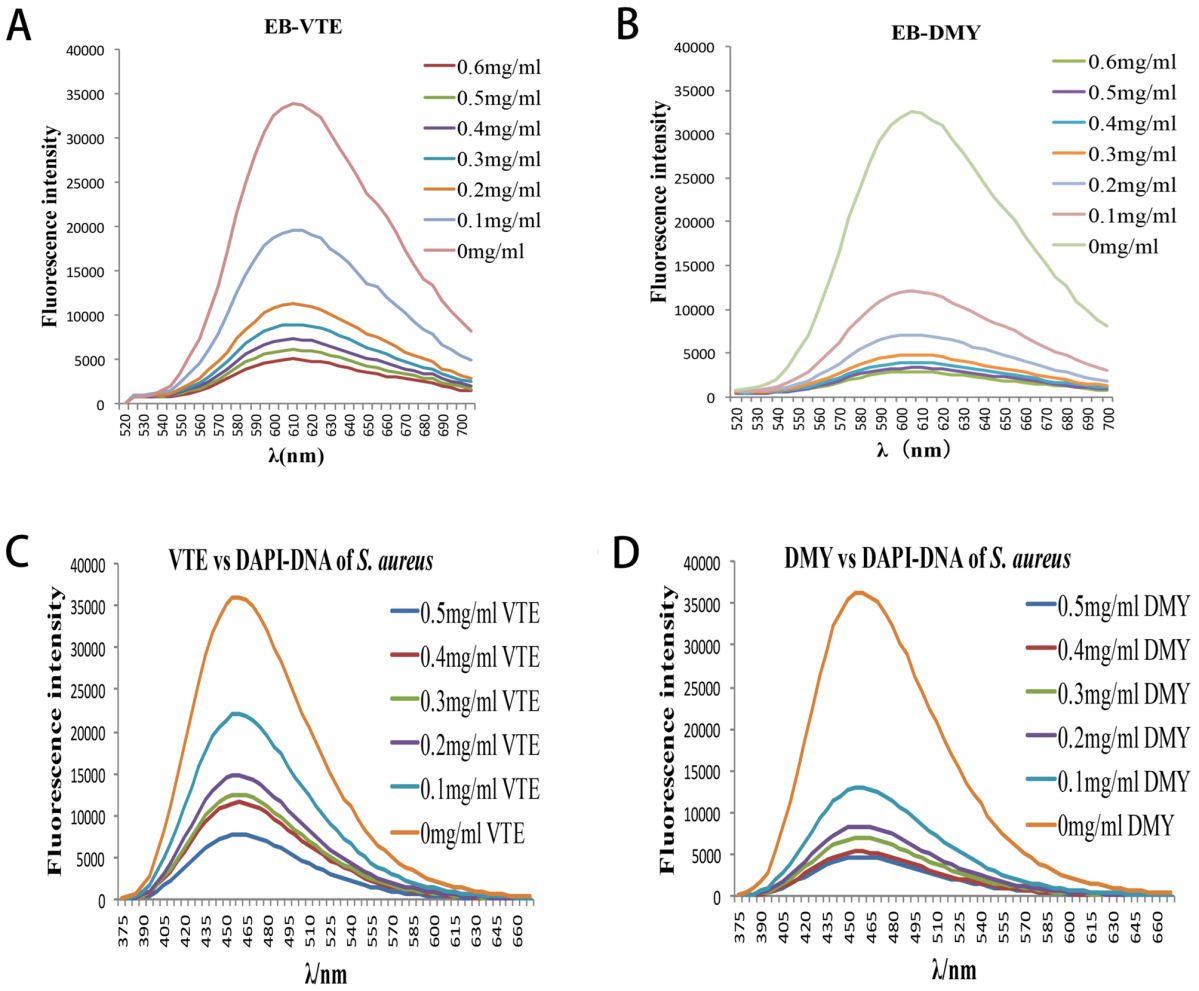
Effect of fluorescence intensity of VTE, DMY versus EB-*S. Aureus* genomic DNA system (**A**,**B**). Effect of fluorescence intensity of VTE, DMY versus DAPI-*S. Aureus* genomic DNA system (**C**,**D**).

The effect of inhibitors on the fluorescence intensity of EB-bacterial genomic DNA system was shown in Fig. 9A,B. After adding different concentrations of VTE and DMY, the maximum emission wavelength of EB-bacterial genomic DNA complex system did not change, and the fluorescence intensity of EB-bacterial genomic DNA system decreased. The fluorescence intensitis of EB- DNA system decreased to less than 60% of the initial fluorescence intensity when the concentrations of VTE and DMY were 0.2 mg/mL and 0.1 mg/mL, respectively. The results show that both VTE and DMY have an insertion binding mode similar to EB. VTE, DMY and EB competed with each other, which replaced most of the EB substances in the DNA system of EB-bacteria^17^.

The effect of inhibitors on the fluorescence intensity of DAPI- bacterial genomic DNA system were shown in Fig. 9C,D. With the addition of VTE and DMY to DAPI-*S.aureus* genomic DNA system, the fluorescence intensity decreased significantly. And the fluorescence intensity of DAPI-*S.aureus* genomic in DNA system decreased by 38.5% and 64.4% when the concentration of VTE and DMY was 0.1 mg/mL, respectively. The results showed that VTE and DMY could replace DAPI and compete for the binding sites of DAPI to bacterial genomic DNA, that is, VTE and DMY combine with the small grooves of the double strands of genomic DNA^18^.

### Antibacterial activity of VTE and DMY in food systems

**Figure 10:**
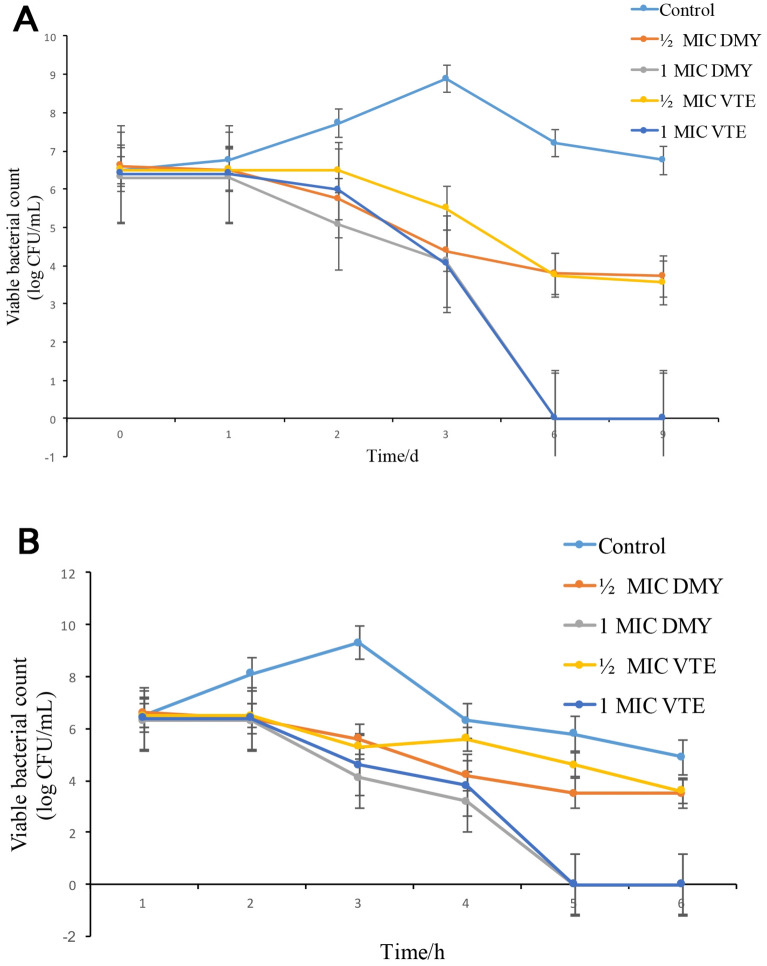
Statistical figure of bacterial colonies in the Challenge of anticorrosion of Chinese Cabbage Juice (**A**). Statistical figure of bacterial colonies in the Challenge of anticorrosion of wort Food (**B**).

As shown in Fig. 10, in the food systems experiment of cabbage juice and wheat juice, 1 × MIC DMY and 1 × MIC VTE could reduce *S.aureus* to 0 in 9 days. On the 6th day, the two inhibitors reduced the colony number to 0 at the same time, and 1/2 × MIC DMY and 1/2 × MIC VTE reduced the colony number from 10^6^ on the 0th day to 10^3^ on the 6th day. The higher concentration of DMY and VTE, the faster number of colonies decreased. The effects of DMY and VTE in different anticorrosive systems were similar, and the colony number was well controlled on the 6th day.

## Discussion

Due to the negative effects of synthetic food additives and the in-depth study of the plant bacteriostatic agents, the research of antibacterial plant extracts as potential food additives is an important direction. The antibacterial activity of flavonoids from plants is being increasingly documented, for example, the MIC of apigenin against *S. aureus* is from 3.9 to 15.6 g/mL^19^. Dihydromyricetin is one of the most abundant flavonoids in VTE with positive antibacterial effect^10^. The results of this study showed that VTE and DMY had antibacterial activity against *S.aureus*, and their MIC were 6.3 mg/mL and 1.25 mg/mL, respectively. Therefore, their antibacterial mechanism was further studied.

Electron microscope experiments can reflect the changes of bacteria caused by VTE and DMY on the whole. So firstly, the whole changes of *S. aureus* treated with VTE and DMY were observed by SEM and TEM. The results of SCE showed that VTE and DMY had effects on the external morphology and structure of the bacteria, and occurred deformation and high bacteriolysis on the surface of the bacteria, especially for VTE. TEM showed that VTE blurred and damaged the cell boundary, and some areas of the nuclear region were whitened. These all showed that VTE and DMY affect the cell wall and nuclear area of *S. aureus*. Moreover, the *S. aureus* treated by VTE and DMY became blurred in cell division, indicating that both VTE and DMY may prevent bacterial division and proliferation by interfering with the formation of cell wall and membrane at the end of cell division. Based on this observation, the mechanism of action of VTE and DMY on the membrane, protein and DNA of *S. aureus* was studied.

Cell wall membrane separates the cell interior from the outside environment and maintains a relatively stable internal environment in which biochemical reactions occur. In this study, classical methods such as AKPase and β-galactosidase were used to detect the integrity and permeability of cell wall and membrane. With the treatment of VTE and DMY, the contents of extracellular AKP and β-galactosidase in the environment were increased, which confirmed the experimental results of membrane destruction. And the effect of VTE on membrane integrity and permeability was stronger than that of DMY (AKPase increased by 21.8% and 10.3%, respectively; The content of extracellular β-galactosidase increased by 90.2% and 57.4%, respectively). Liu^20^ found that DMY could wrinkle the bacteria of Vibrio parahaemolyticus, rough the surface and finally break the cells of Vibrio parahaemolyticus.

Protein synthesis plays an important role in cellular metabolic activity, so the changes of total protein and energy metabolic enzymes were studied. The results showed that the total protein content of *S. aureus* was decreased and shallower of SDS-PAGE bands further confirmed the decrease of total protein synthesis. Next, the activities of some energy metabolic enzymes were also studied. The results showed that the activities of metabolic enzymes (MDH, SDH and total ATPase) were also seriously affected by VTE and DMY, which slowed down the metabolism of bacteria.

DNA regulates macromolecular synthesis and metabolic processes. They play dominant roles in major physiological phenomena including growth, development, and reproduction. Spectral and competitive analysis were used to explore the binding mode of bacterial DNA with VTE and DMY. The results of UV spectra showed that there was more than one interaction between VTE/DMY and DNA. In order to determine the specific binding mode, the fluorescence scanning showed that VTE/DMY interact with DNA by embedding; Competitive analysis further showed that VTE, DMY can compete with EB form intercalation with DNA, then also can compete with DAPI to form groove binding with DNA. In the study of Wu et al. (2017), it was found that DMY could bind to DNA grooves in *S. aureus*, which was verified by the experimental results. However, we found that VTE and DMY still can be embedded with DNA in addition to groove bonding. Compared with the effects of wall, membranes and proteins, the interaction between DMY and DNA was stronger than that of VTE.

In summary, VTE and DMY both had good antibacterial effect on *S. aureus*, and their modes of mechanism were similar. HPLC results showed the DMY was the main component in VTE. However, DMY cannot completely replace VTE in antibacterial. As shown in Table 1, compared with DMY, VTE had a greater effect on bacterial membrane integrity, permeability, protein synthesis and energy metabolic enzyme activity than DMY, while DMY tended to act on internal genetic material DNA. The reason may be that DMY is only the main component of VTE, however there are also myricetin, sinomenine, rutin, quercetin and flavonoid glycosides in VTE.

**Table 1:** The mechanism of VTE and DMY.

Mechanism	VTE (%)	DMY (%)
The extracellular AKPase	+ 21.8	+ 10.3
The extracellular β-galactosidase	+ 90.2	+ 57.4
MDH	− 12.7	− 4.7
SDH	− 65.8	− 16.7
Totle ATPase	− 31.5	− 15.9
The total protein expression	+ 15.5	+ 9.9

The main structure of DMY is 2-phenylbenzo [α] pyran or flavane nucleus: it is composed of two benzene rings (A and B), which are connected by heterocyclic pyran ring (C). Previous studies have shown that the fourth carbonyl oxygen of the C ring, the three-dimensional structure of C–3–OH and C2=C3 on the C ring do not affect the antibacterial activity of flavonoids^18^. It is further reported that the hydroxyl groups on the B ring of flavonoids form hydrogen bonds with DNA bases, or directly inserted into the base to achieve the inhibitory effect on the synthesis of DNA and RNA^19^, our results well supported this inference.

## Conclusion

In this study, it was found that VTE and DMY could inhibit the growth of *S. aureus*. They had similar antibacterial mechanisms. They could destroy the integrity of cell wall and improve the permeability of cell membrane. They also decreased the total protein synthesis and the activities of some energy metabolic enzymes. They could interact with DNA by embedding and grooving bindings. However, the inhibition mode of VTE on *S. aureus* were not exactly the same as DMY. VTE had a greater effect on bacterial membrane integrity, permeability, protein synthesis and energy metabolic enzyme activity. Finally, VTE and DMY also could inhibit the growth of bacteria in barley and cabbage model food system. Additional, they might be used in food or other products for the efficacy of inhibiting bacteria^21,22^. The above results showed that VTE and DMY have great potential as food additives in food industry and so on^23^.
